# Breaking evolutionary and pleiotropic constraints in mammals: On sloths, manatees and homeotic mutations

**DOI:** 10.1186/2041-9139-2-11

**Published:** 2011-05-06

**Authors:** Irma Varela-Lasheras, Alexander J Bakker, Steven D van der Mije, Johan AJ Metz, Joris van Alphen, Frietson Galis

**Affiliations:** 1 NCB Naturalis, Darwinweg1, 2333 CR Leiden, The Netherlands; 2 IIASA, Laxenburg, Austria; 3 VU University Medical Centre, Amsterdam, The Netherlands

## Abstract

**Background:**

Mammals as a rule have seven cervical vertebrae, except for sloths and manatees. Bateson proposed that the change in the number of cervical vertebrae in sloths is due to homeotic transformations. A recent hypothesis proposes that the number of cervical vertebrae in sloths is unchanged and that instead the derived pattern is due to abnormal primaxial/abaxial patterning.

**Results:**

We test the detailed predictions derived from both hypotheses for the skeletal patterns in sloths and manatees for both hypotheses. We find strong support for Bateson's homeosis hypothesis. The observed vertebral and rib patterns cannot be explained by changes in primaxial/abaxial patterning. Vertebral patterns in sloths and manatees are similar to those in mice and humans with abnormal numbers of cervical vertebrae: incomplete and asymmetric homeotic transformations are common and associated with skeletal abnormalities. In sloths the homeotic vertebral shift involves a large part of the vertebral column. As such, similarity is greatest with mice mutant for genes upstream of *Hox*.

**Conclusions:**

We found no skeletal abnormalities in specimens of sister taxa with a normal number of cervical vertebrae. However, we always found such abnormalities in conspecifics with an abnormal number, as in many of the investigated dugongs. These findings strongly support the hypothesis that the evolutionary constraints on changes of the number of cervical vertebrae in mammals is due to deleterious pleitropic effects. We hypothesize that in sloths and manatees low metabolic and activity rates severely reduce the usual stabilizing selection, allowing the breaking of the pleiotropic constraints. This probably also applies to dugongs, although to a lesser extent.

## Background

The seven cervical vertebrae of mammals are remarkably constant in number, regardless of their neck length. In other tetrapods, the number of cervical vertebrae varies considerably and, for instance the long necks of swans consist of 22-25 vertebrae, which provide a flexibility that cannot be matched by the stiff necks of giraffes that consist of seven very long vertebrae [1-3]. In mammals the number of vertebrae in more caudal vertebral regions is also variable, with for example long thoracic regions generally consisting of more vertebrae than short ones [4-6]. In addition to extant mammals, this is also applicable to Mesozoic mammals. For example, *Yanocondon *and *Joholodens *both belong to the same family, but *Yanoconodon *has a longer thoracic region and more thoracic vertebrae than its sister-taxon *Jeholodens *[7].

The constancy of the number of cervical vertebrae probably results from stabilizing selection against changes of that number [8,9]. There we proposed that this selection is indirect and caused by a strong coupling of such changes with deleterious pleiotropic effects, including pediatric cancers. We found support for this hypothesis in a study on early human mortality. Changes of the number of cervical vertebrae were found to be exceptionally common in humans (~7.5% of all conceptions), but strongly selected against: virtually all individuals die before the age of reproduction [[10], see also [11]]. Changes of the number of cervical vertebrae were significantly associated with major congenital abnormalities (deleterious pleiotropic effects). Thus, human data support that pleiotropic constraints [12] are at the root of the evolutionary conservation of the number of cervical vertebrae in mammals. Moreover, we have proposed that the unavoidability of such pleiotropic effects is due to the strong interactions during the early developmental stage when the number of cervical vertebrae is determined [10]. This determination happens as part of the early anterior-posterior patterning of the paraxial mesoderm, mediated by the well-known *Hox *genes [e.g. [13-20]]. The strong interactivety of this early organogenesis stage presumably results from the interactions between the patterning of the three body axes and interactions of these axial patterning processes and simultaneously occurring morphogenetic processes, such as the division and migration of cells, somitogenesis and the active maintenance of the bilateral symmetry of somites [e.g. [10,21-26]]. This strong interactivety leads to a less effective modularity. As a result, slight disturbances of the early organogenesis stage in mammals are frequent and cause deleterious pleiotropic effects [[27], see also [28,29]]. Hence, the low effective modularity not only appears to cause the conservation of the number of cervical vertebrae, but the entire stage. During this stage a large number of traits of the conserved body plan are determined, including the number of limbs, digits, lungs, kidneys, eyes and ears. Therefore, we proposed that pleiotropic constraints and stabilizing selection play a major role in the evolutionary conservation of body plans [30]. Moreover, we argued that a relaxation of this stabilizing selection is necessary to break these pleiotropic constraints so as to allow the evolution of novelties in body plans. An example that shows how a relaxation of stabilizing selection can indeed lead to the persistence of characters against which there is normally strong selection can be found in the evolution of domesticated mammals through artificial selection. Polydactyly, which is strongly evolutionary constrained among amniotes [31,32], is common in many dog breeds with some breed standards even requiring one or two extra toes [31]. Stabilizing selection in dogs is relaxed due to human care and as a result, dogs with many different congenital abnormalities, including polydactyly, can breed and reproduce. Longevity is indeed extremely reduced in many breeds, in particular in large ones, but this does not lead to their extinction [33]. It is presumably this combination of relaxed stabilizing selection and strong directional selection (for changes in size and shape) that has led to the extreme variation in dog shapes, including the presence or absence of extra digits.

### Forward and backward homeosis in the vertebral column

**Figure 1:**
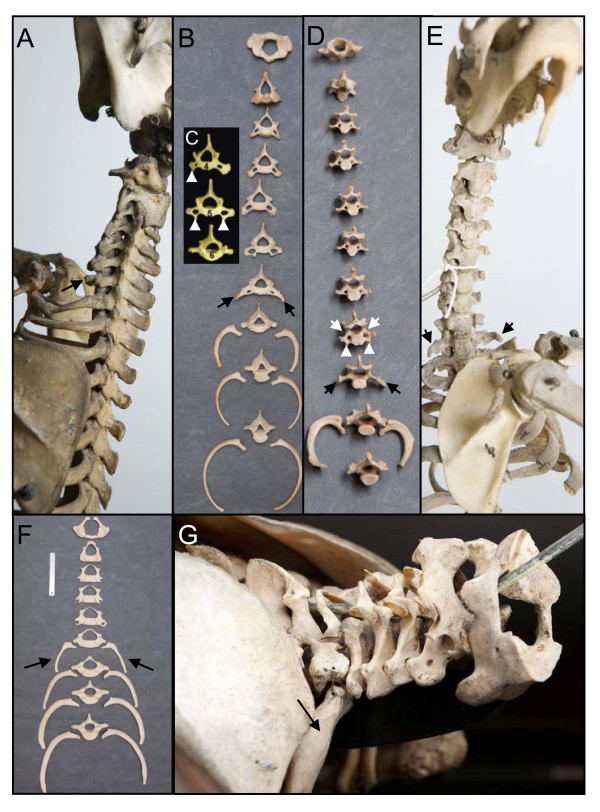
**Sloths and manatees have an abnormal number of cervical vertebrae, which can be seen from the shape of the vertebrae and the absence of ribs**. A and B) *Choloepus didactylus *(ZMA.334 and RMNH.MAM.3274resp.) specimens with six cervical vertebrae and a seventh transitional cervico-thoracic vertebra with rudimentary rib that are fused to the vertebra (arrows). C) Anterior view of the 4^th^, 5^th ^and 6th vertebrae of a *Choloepus hofmanni*. The fourth vertebra has an anterior tuberculum on the right side (white arrowhead) and not on the left, indicating a unilateral homeotic transformation into the 6^th ^cervical vertebra, which is characterized by bilateral tuberculi anterior in mammals. The fifth vertebra has tuberculi anterior bilaterally (white arrowheads), indicating a complete homeotic transformation of the fifth into the sixth cervical vertebra. The 6^th ^vertebra has a completely thoracic shape without foramina transversaria (see Figure 2) and has full ribs, indicating a homeotic transformation into the first thoracic vertebra (normally the 8^th ^vertebra in mammals). Reproduced with permission from [56]. D) and E) *Bradypus tridactylus *(RMNH.MAM.10460 and ZMA.331 resp.) specimens with 8 cervical vertebrae. The 8^th ^vertebra in D) has bilaterally foramina transversaria (white arrow) and tuberculi anterior (white arrowheads), indicating a complete homeotic transformation of the 8^th ^vertebra into the 6^th ^cervical vertebra. In D) and E) the ninth vertebrae have a transitional cervico-thoracic identity with no foramina transversaria and rudimentary ribs that are fused to the vertebrae (arrows). Note the asymmetric length of the ribs of the 10^th ^vertebra. F) anterior view of five cervical vertebrae and four thoracic ones with ribs of *Trichechus manatus *(RMNH.MAM24221). There fifth cervical vertebrae has foramina transversaria, but no tuberculi anterior, as in the transgenic mice with loss of function of *Hoxa5 *[66]). The sixth vertebra has a transitional cervico-thoracic identity with no foramen transversaria, thoracic transverse processes and large cervical ribs (arrows). The seventh vertebra is the first fully thoracic vertebra with full ribs, indicating a complete homeotic transformation. G) Lateral view of *Trichechus senegalensis *(U. Nat coll.) with six cervical vertebrae and a completely thoracic seventh vertebra, with full ribs (arrow), indicating a complete homeotic transformation.

Sloths and manatees are the only mammalian species that as a rule have an exceptional number of cervical vertebrae (Figure 1). Bateson [34] hypothesized that in sloths the exceptional number of cervical vertebrae is due to homeotic transformations of the identity of vertebrae. In the case of *Choloepus *he proposed an anteriorization of cervical and thoracic vertebrae, leading to a cranial shift of the cervico-thoracic boundary and a reduction of the number of cervical vertebrae to six. He coined this "forward homeosis". For *Bradypus *he proposed a posteriorization of cervical and thoracic vertebrae, leading to a caudal shift of the cervico-thoracic boundary and an increase in the number of cervical vertebrae to 8 or 9, which he referred to as "backward homeosis". Furthermore, he postulated that forward and backward homeosis in sloths is not restricted to vertebrae around the cervico-thoracic boundary, but involves the entire vertebral column (see also [35] on a shift of vertebral boundaries in sloths).

Bateson's homeosis hypothesis later obtained support from the discovery that the determination of the number of cervical vertebrae in vertebrates crucially depends on the action of the *Hox *genes. Interspecific and intraspecific changes of this number require homeotic transformations through changes in *Hox *gene expression [e.g. [18,36-40]]. Meristic changes alone cannot explain the change in the number of cervical vertebrae. Even when there are changes in the total number of vertebrae involved, the sequential generation and simultaneous A-P patterning of the somites under control of multiple A-P signaling gradients implies that a change in the number of cervical vertebrae necessarily involves a change of identity of somites around the cervico-thoracic boundary [18,22,24]. Bateson's homeosis hypothesis has been generally accepted and homeotic transformations are also considered to be involved in the abnormal number of cervical vertebrae in manatees [e.g. [6,8,41]]. Similarly, changes in *Hox *gene expression are supposed to be involved in changes of other vertebral regions, e.g. changes of number of rib-bearing vertebrae [19,20] as found in closely related sister-taxa of several entirely extinct Mesozoic mammal groups [7,42]. In this study we test predictions based on Batesons' homeosis hypothesis by investigating whether the vertebral patterns of sloths and manatees are similar to those of mice and other mammals with homeotic mutations.

### Pleiotropic constraints and relaxed selection in sloths

Bateson [34] also surmised that the evolutionary change of the vertebral pattern in sloths must have been associated with pleiotropic effects in neighbouring tissues with important fitness consequences: "this is no trifling thing (...) but on the contrary it effects large portions of the body, each with their proper supply of nerves and blood vessels and the like, producing material change in the mechanics and economy of the whole body, this moreover in wild animals, struggling for their own lives, depending for their existence on the perfection and fitness of their bodily organization" [[34], p. 122]. Such pleiotropic effects in neighbouring tissues are indeed common in humans with a cervical rib (a homeotic change of the seventh cervical vertebra into a thoracic rib-bearing vertebra) and go under the name of Thoracic Outlet Syndrome. This syndrome can lead to serious degeneration of the arm, due to compression of nerves of the brachial plexus, or of blood vessels, by a cervical rib or ligaments and muscles attached to it [e.g. [43-46]].

As mentioned above, the strong interactivity during the determination of the number of cervical vertebrae supposedly leads to even more deleterious pleiotropic effects, resulting in strong prenatal selection of individuals with a changed number of cervical vertebrae. Hence, we expect that it is only thanks to the relaxation of stabilizing selection in sloths and manatees that the pleiotropic constraints against changes of the number of cervical vertebrae could be broken. We have earlier hypothesized that manatees and sloths may indeed experience relaxed selection regimes, associated with their extremely low activity and metabolic rates [8,9,30]. Cancer appears to be one of the deleterious pleiotropic effects associated with a change of the number of cervical vertebrae in humans, high childhood cancer rates (~120 fold increase) were found to be associated with the presence of cervical ribs (a rib on the seventh cervical vertebra, representing a homeotic change of the seventh vertebra into a thoracic one [47,48]. Low metabolic rates are associated with low oxidative DNA damage and due to this presumably with low cancer rates [49-52]. Hence, we hypothesized that in sloths and manatees cancer rates may be low and, thus, cause less stabilizing selection against changes of the number of cervical vertebrae. In agreement with this, cancer rates in manatees indeed appear to be low [9]. The low activity rates are also thought to diminish or prevent more direct harmful side effects of cervical ribs observed in humans, c.q. Thoracic Outlet Syndrome. The severity of this syndrome has been found to be particularly strong in athletes and appears to be positively associated with strenuous activity [8,53-55]. In view of the extremely low activity rates of sloths and manatees direct pleiotropic effects in neighbouring tissues, such as a slightly changed position of ligaments, muscles, blood vessels or nerves, may have little or no effect on their fitness.

In this study, we further investigate the hypothesis that the number of cervical vertebrae in mammals is conserved by pleiotropic constraints and that these constraints have been broken in sloths and manatees due to the relaxation of stabilizing selection. To this end we compare the vertebral patterns and other skeletal characteristics in sloths and manatees with those of several sistertaxa, to wit anteaters, armadillos, dugongs and hyraxes.

### An alternative hypothesis for the exceptional cervical vertebrae in sloths: aberrant primaxial/abaxial patterning

**Figure 2:**
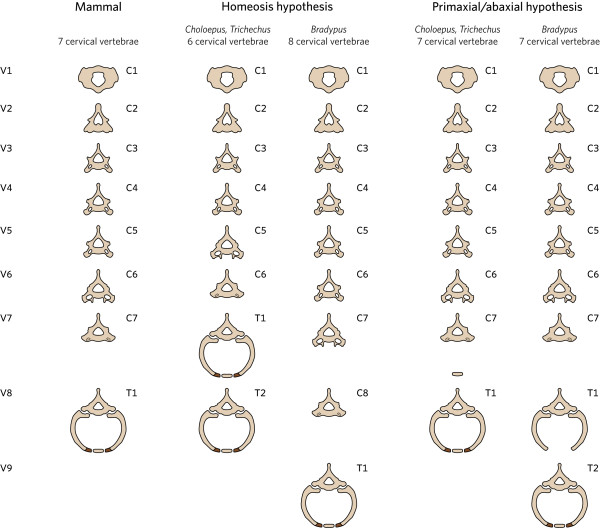
**Schedule to show the predictions for vertebebrae shape and the presence or absence of ribs of the Homeosis hypothesis and Primaxial/abaxial hypothesis for sloths (*Choloepus, Bradypus*) and manatees (*Trichechus*)**. The sternal rib parts are indicated by darker coloration. Note that the sternal rib parts are usuall small in the first and second ribs of mammals.

Recently Buchholtz and Stepien [56] proposed an alternative hypothesis for the derived pattern of cervical vertebrae in sloths. They reject the homeosis hypothesis of Bateson [26]. Instead they claim that the identity of the first seven vertebrae, as defined by the primary *Hox *pattern, is cervical and of the adjacent more posterior ones, thoracic, as in other mammals. In other words, they assume that the number of cervical vertebrae has not been changed at all. Instead they propose that the anterior number of vertebrae without full ribs has changed, without an associated change of the identity of the vertebrae (Figure 2). Moreover, they propose that the abnormal number of vertebrae without full ribs in sloths is caused by a shift of the abaxial domain (lateral plate mesoderm and somatic cells that migrate in the lateral plate mesoderm [57], relative to the adjacent and stationary primaxial domain (paraxial mesoderm without the migrating paraxial mesoderm cells [57]). Thus, Buchholtz and Stepien [56] imply that changes in the interactions between lateral plate mesoderm and paraxial mesoderm cells that migrate into the lateral plate mesoderm, so-called primaxial/abaxial patterning, lead to changes in the presence or absence of the sternal rib components, which are the distalmost rib components, closest to the sternum (Figure 2). The prediction that sternal rib parts are induced due to a change in abaxial signaling requires that this signaling is *instructive *(initiating). This in contrast to a *permissive *(guiding) abaxial signalling towards sternal rib parts.

### Abaxial signalling is not instructive

The prediction that sternal rib parts are induced due to a change in abaxial signalling requires that this signalling is *instructive*. This in contrast to a *permissive *abaxial signalling towards sternal rib parts. Theoretically, the instructive patterning by the lateral plate mesoderm of a meristic series of sternal rib components, independently and in the absence of the normally present meristic series of vertebral rib components, seems highly unlikely. Furthermore, experimental evidence shows that the signalling by the abaxial domain is necessary for the development of sternal rib parts [e.g. [58-62]]; however, this signalling appears to be permissive rather than instructive [[59], see also [63]]. Chevallier [59] demonstrated with transplantation experiments on chickens and quails, that only somites that normally contribute to ribs with sternal parts (i.e. the most caudal thoracic somites) can develop sternal components, provided they are grafted close enough to the developing sternum. Furthermore, he showed that thoracic somites that normally contribute to ribs without these sternal parts (i.e. the most anterior thoracic somites), never develop sternal parts, even not when transplanted to the thoracic region where ribs normally develop them. In support of this, Jacob et al. [63] found that after grafting of unsegmented mesoderm of the anterior thoracic region into the hindlimb bud, four ribs developed, the two most anterior ones without and the two more posterior ones with sternal rib-like parts (see Figure 5 of Jacob et al. [63]). This shows that abaxial signalling is necessary for the formation of sternal rib parts, but permissive, and not instructive. Furthermore, it shows that the permissive signalling is also present in the hindlimb bud. In further support of the permissive nature of abaxial signalling, transplantation of cervical and lumbar paraxial mesoderm to the thoracic region never resulted in the formation of sternal rib parts, nor of any other rib parts [57,58]. The permissive nature of this abaxial signalling in chickens is further in agreement with the findings of Smith et al. [61] on mice, that the specification of the sternal ribs parts occurs before the penetration of the sternal rib anlage into the lateral plate mesoderm (abaxial domain) and, hence, before primaxial/abaxial patterning. In addition, it is questionable whether the development of sternal rib components is possible in the complete absence of the development of the adjacent vertebral rib components [58,64,65]. In conclusion, there is only empirical evidence that instructive signalling for the formation of ribs, including sternal rib parts, occurs in the paraxial mesoderm, mediated by *Hox *genes [[59,63-65] see also [40]]. Recent data of Vinagre et al [20] emphasizes this conclusion, as they found that *Hox *specification of the rib-containing region of the vertebral column is mediated by *Myf5 *and *Myf6 *activity in the myotomal part of the somites. There is no empirical support for instructive abaxial signalling for the formation of sternal, or other rib parts in the literature. These developmental insights from chickens and mice, if applicable for sloths, tell that a shift of the abaxial domain relative to the stationary primaxial domain cannot explain the presence of full or rudimentary ribs on cervical or lumbar vertebrae. Similarly, a shift of the abaxial domain cannot explain the complete absence of ribs in thoracic vertebrae. Such a shift of the abaxial domain can only explain the absence of sternal rib parts in ribs of thoracic vertebrae. Hence, the assumption of Buchholtz and Stepien [56] for instructive rather than permissive abaxial signalling of sternal rib parts is not supported by empirical evidence and this has implications for the predictions of the primaxial/abaxial hypothesis.

### Testing the support for the homeosis and primaxial/abaxial hypotheses

It is important to investigate the strength of the support for the two radically different hypotheses, to better understand the role of development and developmental constraints in the evolution of body plans. To this aim we formulate predictions based on the two hypotheses and we have test these predictions by investigating skeletal patterns in wild-caught specimens of sloths. In addition, we investigate the skeletal patterns of manatees in the light of these hypotheses. Finally, we compare the skeletal patterns of sloths and manatees with related taxa, to wit anteaters, armadillos, dugongs and hyraxes.

## Methods

### Specimens

Folivora (sloths). We analysed skeletons of 16 *Choloepus didactylus *(L.) specimens of the NCB Naturalis, Leiden, of 3 *Choloepus hoffmanni *(Peters) specimens of the Royal Belgian Institute, Brussels and of 11 *Bradypus tridactylus *(L.) specimens of the NCB Naturalis.

Dasypodidae (armadillos). We analysed skeletons of 9 *Dasypus novemcinctus *(L.), 12 *Euphractus sexcinctus *(L.), 1 *Dasypus kappleri *(Krauss), specimens, 2 *Cabassous unicinctus *(L.), 1 *Chaetophractus vellerosus *(Gray), 1 *Chaetophractus villosus *(Desmarest), 1 *Priodontes maximus *(Kerr) specimens of the NCB Naturalis.

Myrmecophagidae (anteaters). We analysed skeletons of 8 *Tamandua tetradactyla *(L.) specimens of the Royal Belgian Institute, Brussels.

Procaviidae (hyraxes). We analysed skeletons of 7 *Dendrohyrax dorsalis *(Fraser) specimens, 5 of the Royal Museum of Central Africa, 2 of the NCB Naturalis, 4 *Dendrohyrax arboreus *(Smith), 2 of the NCB Naturalis, 2 of the Royal Museum of Central Africa, 6 *Procavia capensis*(Pallas), 4 of the NCB Naturalis, 2 of the Royal Museum of Central Africa.

Trichechidae (manatees). We analysed skeletons of 13 *Trichechus manatus *(L.) specimens of the NCB Naturalis and 5 *Trichechus senegalensis *(Link) specimens of which 2 of the NCB Naturalis, 2 of the Royal Museum of Central Africa, Tervuren and 2 of the Natural History Museum, London.

Dugongidae (Dugongs). We analysed skeletons of 11 *Dugong dugon *(Müller) speciments, 4 from the Royal Belgian Institute, 4 from the Natural History Museum London and 3 from the NCB Naturalis.

All specimens are listed with vertebral formula and abnormalities in Tables 1,2,3,4,5,6.

**Table 1:** Vertebral information and congenital abnormalities in investigated mammalian specimens: Folivorea (sloths)

Folivora (sloths)							
**Collection**	**Species**	**Collection No**.	**Sex**	**Vertebral formula**	**Presacral No**.	**Rud. ribs on vertebrae**	**Skeletal and fibrous abnormalities**

		15260 **)	F	5C 1C/T 22T 1T/L 3L 6S 1S/Cg 5Cg	32	V6, V29	C2/C3 fusion, deformation of pelvic girdle, hole in scapula
		
RBINC	*Choloepus hoffmanni*	16349 **)	F	5C 1C/T 21T 1T/L 3L 1L/S 5S 5Cg	31.5	V6	C1/C2 fusion, hole in larynx
		
		16348 **)	F	5C 1C/T 21T 4L 1L/S 7S 1SCg 4Cg	31.5	V6	C2/C3 fusion

		RMNH.MAM.322 **)	F	6C 1C/T 23T 4L 7S 1S/Cg 5Cg	34	V7	Malformed first ribs, hole in scapula
		
		RMNH.MAM.3961	F	6C 1C/T 23T 1T/L 2L 1L/S 7S 5Cg	33.5	V7	-
		
		RMNH.MAM.24470	n.a.	5C 2C/T 24T 4L 7S 1S/Cg 3+Cg	35	V6,V7	Abnormal fibrous band attached to rudimentary rib
		
		RMNH.MAM.24469	n.a.	5C 2C/T 23T 4L 7S 4Cg	34	V6 V7	Incomplete ossification sternum
		
		RMNH.MAM.3274	n.a.	6C 1C/T 22T 1T/L 4L 7S 6Cg	34	V7	-
		
		RMNH.MAM.3465	n.a.	5C 2C/T 23T 1T/L 3L 7S 5Cg	34	V6 V7	C2-C3 fusion, ossification absent in sternum, fusion of ribs on V6-V7,
		
		RMNH.MAM.1002 **)	F	5C 2C/T 23T 3L 1L/S 7S 4Cg	33.5	V6 V7	C2-C3 fusion, deformation of first ribs, asymmetric sternum, fusion of rudimentary ribs on V6-V7
		
NCBN	*Choloepus didactylus*	RMNH.MAM.7203 **)	F	6C 1C/T 23T 4L 7S 1S/Cg +Cg	34	V7	C2-C3 fusion, V7-V8 fusion, asymmetric vertebrae, fibrous band attached to rudimentary ribs
		
		RMNH.MAM.2552	F	6C 1C/T 24T 3L 7S 1S/Cg 5Cg	34	V7	Malformed ribs (3 most anterior ones)
		
		RMNH.MAM.1673	F	6C 1C/T 24T 3L 8S 5Cg	34	V7	C3,C4,C5 fused
		
		ZMA335	n.a.	6C 1C/T 24T 3L 8S 4CG	34	V7	Ossification absent in sternum and sacrum
		
		ZMA334	n.a.	5C 2C/T 23T 4L 1L/S 9S 3+Cg	34.5	V6	C2-C3 fusion
		
		ZMA.336	n.a.	6C 1C/T 1C/T 22T 4L 8S 4Cg	34	V7 V8	Abnormal fibrous band attached to rudimentary rib, incomplete ossification
		
		RMNH.MAM.11417	F	-	-	-	Malformed humeri, oligodontia
		
		RMNH.MAM.1156	n.a.	1L 1L/S 7S 4Cg	-	-	*)
		
		ZMA.9765	F	6S 1S/Cg	-	-	*)
		
		RMNH.MAM.21576	n.a.	8C 1C/T 15T 3L 1L/S 6S 9Cg	28	V9	Asymmetric cranium
		
		RMNH.MAM.21581	n.a.	7C 2C/T 14T 1T/L 3L 7S 8Cg	27	V8 V9 V24	-
		
		RMNH.MAM.24440	n.a.	8C 1C/T 16T	-	-	Asymmetric cranium
		
		RMNH.MAM.10460	F	8C 1C/T 15T 4L 6S 8Cg	28	V9	Irregularly shaped first rib
		
		RMNH.MAM.10459	F	8C 1C/T 14T 3L 1L/S 6S 6C 6+Cg	27	V9	Metacarpal, metatarsal anomalies, many fractures, hole in cranium (unknown cause)
		
	*Bradypus tridactylus*	RMNH.MAM.18781	F	8C 1C/T 14T 4L 6S 8Cg	27	V9	-
		
		RMNH.MAM.24421	n.a.	8C 1C/T 15T 4L 5S 10Cg	28	-	-
		
		ZMA331	n.a.	7C 2C/T 14T 1T/L 4L 6S 1S/Cg 7Cg	28	V8 V9 V24	Irregularly shaped vertebrae and first rib
		
		ZMA332	n.a.	8C 1C/T 15T 1T/L 3L 6S 1S/Cg 6+Cg	28	V9 V25	-
		
		ZMA924 **)	n.a.	8C 1C/T 15T 3L 6S 7Cg	27	V9	Irregularly shaped vertebrae and first rib
		
		U. ZMA coll. **)	n.a.	8C 1C/T 15T 4L 5S 1S/Cg 8Cg	28	V9	-

*) Skeleton largely absent

**) Died in captivity

Abbreviations: n.a. - not available, C -Cervical, T - Thoracic, L-Lumbar, S - Sacral, Cg - Coccygeal, Cd - Caudal (post thoracic), V - Vertebra, rud. - rudimentary, NCBN - NCB Naturalis, RMCA - Royal Museum for Central Africa Tervuren, RBINC - Royal Belgian Institute of Natural Sciences Brussels, NHM - Natural History Museum London

**Table 2:** Vertebral information and congenital abnormalities in investigated mammalian specimens: Trichechidae (manatees)

Trichechidae (manatees)							
Collection	Species	**Collection No**.	Sex	Vertebral formula	**Precaudal No**.	Rud. ribs on vertebrae	Skeletal and fibrous abnormalities
RBINC		1,181	n.a.	6C 17T 1T/Cd 26Cd	23.5	V24	V7-V8 fusion, sternal foramen and asymmetric sternum.
		
		U. Nat coll.	n.a.	5C 2C/T 17T 2+Cd	24	V6, V7	Abnormal fibrous band sfrom rudimentary rib to sternum and rostral to sternum, sternal foramen
		
		RMNH.MAM.16050	M	4C 2C/T 17T 26Cd	23	V5, V6	Asymmetric sternum, cervical spinous processes reduced and not fused
		
		RMNH.MAM.24221 **)	F	5C 1C/T 17T 1T/Cd 24Cd	23.5	V6, V24	Sternal foramen and asymmetric sternum, cervical spinous processes reduced and not fused
		
		RMNH.MAM.16049	M	5C 1C/T 16T 1T/Cd 26Cd	22.5	V6, V23	-
		
		RMNH.MAM.22392	F	4C 2C/T 16T 1T/Cd 26Cd	22.5	V5, V6, V23	Sternal foramen and asymmetric sternum, malformed cervical vertebrae
		
	*Trichechus manatus*	ZMA.10725	n.a.	5C 1C/T 17T 25Cd	23	V6	Sternal foramen and asymmetric sternum and asymmetric pelvic rudiments
		
NCBN		ZMA.9550	n.a.	5C 1C/T 16T 17+Cd	22	V6	C2-C3 fusion, malformed cervical vertebrae
		
		ZMA.9549	F	5C 1C/T 16T 1T/Cd 26Cd	22.5	V6, V23	Sternal foramen and asymmetric sternum, malformed caudal ribs
		
		ZMA.23909 **)	n.a.	5C 1C/T 16T 1T/Cd 25Cd	22.5	V6,V23	Sternal foramen and asymmetric sternum
		
		ZMA.1342 **)	M	5C 1C/T 15T 24Cd	21	V6	Sternal foramen, malformed cervical vertebrae
		
		ZMA.1340	n.a.	5C 1C/T 17T 23+Cd	23	V6	Abnormal fibrous band from rudimentary rib to sternum and rostral to sternum
		
		ZMA.26773	n.a.	5C 1C/T 17T 1T/Cd 13+Cd	23.5	V6, V24	Asymmetric sternum
	
		U. Nat coll.	n.a.	6C 17T 2Cd	23	-	C2-C3 fusion, asymmetric sternum.
		
		ZMA.14042	M	5C 1C/T 17T 26Cd	23	V6	C2-C3 fusion, asymmetric sternum.
		
NHM	*Trichechus senegalensis*	1999.77	n.a.	6C 16T	-	-	C2-C3 fusion, asymmetric ribs, deformation of scapula and limbs
		
		1864.12.1	n.a.	5C 1C/T 15T 1T/Cd 26Cd	21.5	V6, V22	C2-C3 fusion
		
RMCA		21530	F	5C 1C/T 17T 27Cd	23	V6	C3-C4 fusion.

*) Skeleton largely absent

**) Died in captivity

Abbreviations: n.a. - not available, C -Cervical, T - Thoracic, L-Lumbar, S - Sacral, Cg - Coccygeal, Cd - Caudal (post thoracic), V - Vertebra, rud. - rudimentary, NCBN - NCB Naturalis, RMCA - Royal Museum for Central Africa Tervuren, RBINC - Royal Belgian Institute of Natural Sciences Brussels, NHM - Natural History Museum London

**Table 3:** Vertebral information and congenital abnormalities in investigated mammalian specimens: Myrmecophagidae (Anteaters)

Myrmecophagidae (Anteaters)							
Collection	Species	**Collection No**.	Sex	Vertebral formula	**Presacral No**.	Rud. ribs on vertebrae	Skeletal and fibrous abnormalities
		17277 **)	F	7C 17T 1T/L 3L 5S 31+Cg	28	V25	
		
		313 beta **)	F	7C 17T 3L 5S 32Cg	27	-	Some irregularly shaped tail vertebrae
		
		312 delta **)	-	7C 17T 3L 5S 35Cg	27	-	
		
		15641 **)	F	6C 1C/T 16T 1T/L 1L 1L/S 5S 1S/Cg 35Cg	25.5	V7	Incomplete ossification sternum, oligodactyly
		
RBINC	*Tamandua tetradactyla*	8986 **)	F	7C 17T 1T/L 1L 1L/S 5S 41Cg	26.5	V25	Some irregularly shaped tail vertebrae
		
		7886 **)	F	7C 17T 3L 6S 39Cg	27	-	Irregularly shaped tail vertebra
		
		314 delta **)	M	7C 16T 3L 5S 1S/Cg 35Cg	26	-	
		
		312 gamma **)	-	7C 17T 3L 1L/S 5S 37Cg	27.5	-	

*) Skeleton largely absent

**) Died in captivity

Abbreviations: n.a. - not available, C -Cervical, T - Thoracic, L-Lumbar, S - Sacral, Cg - Coccygeal, Cd - Caudal (post thoracic), V - Vertebra, rud. - rudimentary, NCBN - NCB Naturalis, RMCA - Royal Museum for Central Africa Tervuren, RBINC - Royal Belgian Institute of Natural Sciences Brussels, NHM - Natural History Museum London

**Table 4:** Vertebral information and congenital abnormalities in investigated mammalian specimens: Dasypodidae (Armadillos)

Dasypodidae (Armadillos)							
**Collection**	**Species**	**Collection No**.	**Sex**	**Vertebral formula**	**Presacral No**.	**Rud. ribs on vertebrae**	**Skeletal and fibrous abnormalities**

		RMNH.MAM.11373 **)	F	7C 10T 5L 9S 19+Cg	22	-	-
		
		RMNH.MAM.20966	M	7C 11T 5L 9S 2+Cg	23	-	-
		
		RMNH.MAM.20968	F	7C 10T 6L 9S 2+Cg	23	-	-
		
		RMNH.MAM.20969	M	7C 11T 5L 8S 21Cg	23	-	-
		
	*Dasypus novemcinctus*	RMNH.MAM.21029	n.a.	7C 11T 5L 9S 20+Cg	23	-	-
		
		RMNH.MAM.21030	n.a.	7C 11T 5L 8S 1S/Cg 23Cg	23	-	-
		
		RMNH.MAM.21031	n.a.	7C 11T 5L 9S 21Cg	23	-	-
		
		ZMA.314	n.a.	7C 11T 5L 1L/S 8S 14+Cg	23.5	-	-
		
		ZMA.313	n.a.	7C 10T 5L 8S 1S/Cg 21Cg	22	-	-
	
		RMNH.MAM.4159 **)	M	7C 11T 3L 8S 18+Cg	21	-	-
		
		RMNH.MAM.2249	F	7C 11T 3L 8S 25Cg	21	-	-
		
		RMNH.MAM.21036	n.a.	7C 11T 3L 8S 17+Cg	21	-	-
		
		RMNH.MAM.20527	M	7C 11T 3L 8S 21+Cg	21	-	-
		
NCBN		RMNH.MAM.20528	M	7C 11T 3L 8S 19+Cg	21	-	-
		
	*Euphractus sexcinctus*	RMNH.MAM.21037 **)	M	7C 11T 3L 8S 17+Cg	21	-	-
		
		U. Nat. Coll. **)	F	7C 11T 3L 8S 17+Cg	21	-	-
		
		U. Nat. Coll. **)	F	7C 11T 3L 8S 18+Cg	21	-	-
		
		ZMA.325	n.a.	7C 11T 3L 8S 8+Cg	21	-	-
		
		ZMA.7314	n.a.	7C 11T 3L 1L/S 7S 1S/Cg 14+Cg	21.5	-	-
		
		ZMA.326	n.a.	7C 11T 3L 9S 12+Cg	21	-	-
		
		ZMA.324	n.a.	7C 11T 4L 8S 15+Cg	22	-	-
	
	*Dasypus kappleri*	RMNH.MAM.20965	n.a.	7C 9T 5L 8S 18+Cg	21	-	-
	
	*Chaetophractus vellerosus*	RMNH.MAM.21038 **)	F	7C 11T 3L 8S 15+Cg	21	-	-
	
	*Chaetophractus villosus*	RMNH.MAM.2533	M	7C 11T 1T/L 2L 1L/S 7S 1+6Cg	21.5	V19	-
	
		RMNH.MAM.21039	n.a.	7C 13T 1T/L 3L 1L/S 10S 16+Cg	24.5	V21	-
		
	*Cabassous unicinctus*	RMNH.MAM.21040	M	7C 12T 3L 9S 19Cg	22	-	-
	
	*Priodontes maximus*	ZMA.323	n.a.	7C 11T 4L 12S 21Cg	22	-	-

*) Skeleton largely absent

**) Died in captivity

Abbreviations: n.a. - not available, C -Cervical, T - Thoracic, L-Lumbar, S - Sacral, Cg - Coccygeal, Cd - Caudal (post thoracic), V - Vertebra, rud. - rudimentary, NCBN - NCB Naturalis, RMCA - Royal Museum for Central Africa Tervuren, RBINC - Royal Belgian Institute of Natural Sciences Brussels, NHM - Natural History Museum London

**Table 5:** Vertebral information and congenital abnormalities in investigated mammalian specimens: Dugongidae (Dugongs)

Dugongidae (Dugongs)							
Collection	Species	**Collection No**.	Sex	Vertebral formula	**Precaudal No**.	Rud. ribs on vertebrae	Skeletal and fibrous abnormalities
		ZMA.8870	n.a.	6C 1C/T 19T 25+Cd	26	V7	Malformed cervical vertebrae
		
NCBN		RMNH.MAM.27522	n.a.	7C 19T 1T/Cd 34Cd	26	-	-
		
		RMNH.MAM.27523	n.a.	6C 1C/T 19T 1T/Cd 33Cd	26.5	V7, V27	C2-C3 fusion, rib fusions, malformed cervical vertebrae, fusion of 4th and 5th digit of forelimb
		
		1946.8.6.1	F	6C 1C/T 19T 24+Cd	26	V7	Asymmetric sternum, malformed vertebrae, tiny hole in cranium (midline)
		
		1870.8.16.1	M	7C 19T 30Cd	26	-	-
		
NHM	*Dugong dugon*	1966 9.7.1	M	6C 1C/T 19T 29Cd	26	V7	Cranium, vertebrae and ribs irregularly shaped, asymmetric sternum
		
		1885.4.20.2	F	6C 1C/T 18T 1T/Cd 31Cd	25.5	V7, V26	Cranium and sternum malformed
		
		1.183d	n.a.	6C 1C/T 19T 35Cd	26	V7	Asymmetric sternum, abnormal ossification of tendons in manus, arthrosis, severe osteoporosis
		
RBINC		800B1/2	F	7C 18T 1T/Cd 31Cd	25.5	V26	-
		
		800	n.a.	7C 18T 26+Cd	25	-	Tiny hole in cranium (midline)
		
		1.183B	n.a.	7C 18T 33Cd	25	-	Tiny hole in cranium (midline)

*) Skeleton largely absent

**) Died in captivity

Abbreviations: n.a. - not available, C -Cervical, T - Thoracic, L-Lumbar, S - Sacral, Cg - Coccygeal, Cd - Caudal (post thoracic), V - Vertebra, rud. - rudimentary, NCBN - NCB Naturalis, RMCA - Royal Museum for Central Africa Tervuren, RBINC - Royal Belgian Institute of Natural Sciences Brussels, NHM - Natural History Museum London

**Table 6:** Vertebral information and congenital abnormalities in investigated mammalian specimens: Procaviidae (Hyraxes)

Procaviidae (Hyraxes)							
Collection	Species	**Collection No**.	Sex	Vertebral formula	**Presacral No**.	Rud. ribs on vertebrae	Skeletal and fibrous abnormalities
		RMNH.MAM.2150	F	7C 22T 7L 4S 1S/Cg 7Cg	36	-	-
		
NCBN		RMNH.MAM.45276	F	7C 20T 9L 4S 7Cg	36	-	-
		
		83	n.a.	21T 8L 3S 1S/Cg 4+Cg	-	-	-
		
	*Dendrohyrax dorsalis*	15906	n.a.	7C 21T 7L 1L/S 3S 2S/Cg 3+Cg	35.5	-	-
		
RMCA		17255	n.a.	7C 20T 1T/L 6L 1L/S 4S 6+Cg	34.5	V28	-
		
		26676 **)	n.a.	7C 20T 7L 6S 1S/Cg 5+Cg	34	-	-
		
		28795	M	7C 20T 1T/L 8L 5S 1S/Cg 1+Cg	36	-	-

NCBN		RMNH.MAM.45277	M	7C 20T 1T/L 7L 1L/S 4S 7Cg	35.5	-	-
		
	*Dendrohyrax arboreus*	RMNH.MAM.45278	M	7C 21T 7L 1L/S 5S	35.5	-	-
		
		22057	M	6C 20T 1T/L 9L 6S +Cg	36	V27	Oligodactyly
		
RMCA		3590	n.a.	7C 21T 7L 6S 1+Cg	35	-	-

		RMNH.MAM.45279	n.a.	7C 21T 9L 4S 8Cg	37	-	-
		
		RMNH.MAM.45280	F	7C 21T 9L 4S 7Cg	37	-	-
		
NCBN		ZMA.306	n.a.	7C 22T 8L 4S 7Cg	37	-	-
		
	*Procavia capensis*	ZMA.307	n.a.	7C 21T 7L 1L/S 4S 8Cg	35.5	-	-
		
		35315	F	Q7C 21T 8L 6S 4+Cg	36	-	-
		
RMCA		20098	n.a.	6C 1C/T 20T 1T/L 8L 6S 4+Cg	36	V28	Malformations of vertebral column and scapulae

*) Skeleton largely absent

**) Died in captivity

Abbreviations: n.a. - not available, C -Cervical, T - Thoracic, L-Lumbar, S - Sacral, Cg - Coccygeal, Cd - Caudal (post thoracic), V - Vertebra, rud. - rudimentary, NCBN - NCB Naturalis, RMCA - Royal Museum for Central Africa Tervuren, RBINC - Royal Belgian Institute of Natural Sciences Brussels, NHM - Natural History Museum London

### Vertebral formula

We have determined the vertebral formula of skeletons of the selected mammalian specimens by determining the number of cervical, thoracic, lumbar, sacral and coccygeal vertebrae, as far as present. In the case of manatees and dugongs only the number of cervical, thoracic and caudal vertebrae was determined.

### Diagnosis of congenital abnormalities

All skeletons were analysed for the presence of congenital abnormalities, such as chondrification and ossification defects, absent or malformed skeletal parts, supernumerary skeletal parts, midline fusions defects and ectopic fibrous bands.

### Testing of hypotheses

We formulated predictions to test the strength of the support for the homeosis versus the primaxial/abaxial hypothesis. We based our predictions on knowledge of development of vertebrae and ribs as revealed by published experiments on mice and chickens. In addition, we based the predictions on knowledge of homeotic transformations of vertebrae as revealed by phenotypic variation found in transgenic homeotic mice mutants and in natural homeotic mutants in other mammalian species, including humans.

We tested the predictions by analysing the skeletal patterns of sloths and manatees and for comparison those of sistertaxa of which the number of cervical vertebrae was expected to be seven, i.e. anteaters, armadillos for sloths and dugongs and hyraxes for manatees.

## Comparing the homeosis and priaxial/abaxial hypotheses for sloths

### Predictions

#### Number and shape of cervical vertebrae

**Figure 3:**
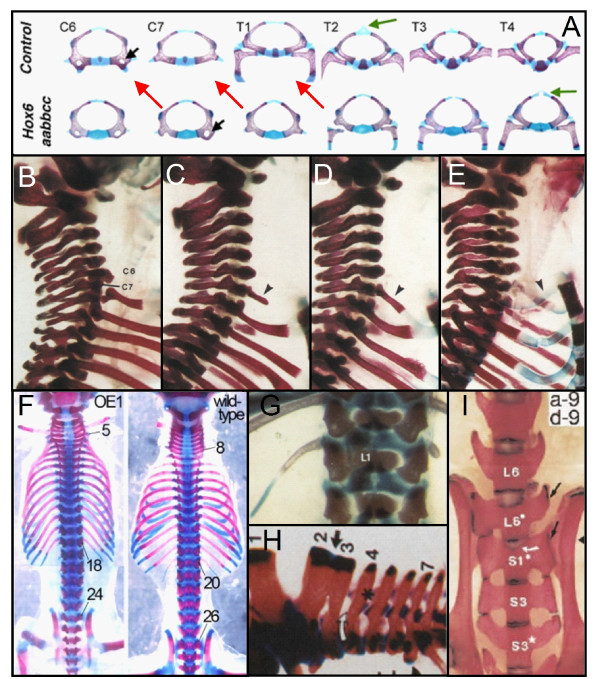
**Vertebrae in transgenic mice with homeotic mutations**. A) homeotic transformations of vertebrae in *Hox6 *paralogous mutants (red arrows) with 8 cervical vertebrae instead of 7. Reproduced with permission from [39]. B), C), D), E) showing incomplete and complete homeotic transformations in mice with loss of function of Hoxa5. B) wild-type, C) short rudimentary rib on C7 (arrowhead), D) large rudimentary rib on C7 which is fused to the first thoracic rib (arrowhead), E) complete rib on C7 which is fused to the sternum (arrowhead). Reproduced with permission from [66]3. F) Change in the number of presacral vertebrae from 25 to 23 in transgenic mice with overexpression of *Cdx1*. Reproduced with permission from [97]. G) Asymmetric lumbar ribs in transgenic mice with loss of function of *Hoxc8*. Reproduced with permission from [138]. H) Fusion of the spinous processes of the second and third cervical vertebrae in a *Hoxa4-b4 *double mutant. Reproduced with permission from [138] I) Asymmetric and transitional lumbo-sacral vertebra with incomplete fusion of L6 to the sacrum (arrow). Reproduced with permission from [139].

The homeosis hypothesis postulates that in sloths the number of vertebrae with a cervical identity is changed compared to mammals with seven cervical vertebrae, i.e., the number of anterior vertebrae without ribs, as well as the shape of the vertebrae around the cervico-thoracic boundary is changed (Figure 2). Hence, the prediction for *Bradypus *is that more vertebrae will have cervical shape characteristics, such as the presence of foramina transversaria and more vertebrae will not have ribs (Figure 2). In addition, it is expected that the anterior tubercles, which normally are largest on the sixth vertebra, will be largest on a more caudal vertebra. The reverse is predicted for *Choloepus *specimens, i.e. they will have fewer vertebrae with cervical shape characteristics, coinciding with the absence of ribs (Figure 2). This is in agreement with shape and rib changes in homeotic mice mutants with fewer or more than seven cervical vertebrae (Figure 3A, [e.g. [39,66-70]].

The primaxial/abaxial hypothesis postulates that in *Bradypus *and *Choloepus *the first seven vertebrae have a cervical identity, which then should be visible in their shape characteristics (Figure 2). In addition, for *Bradypus *it predicts that the ribs of the 8^th ^and possibly the 9^th ^vertebra will only possess proximal and medial (vertebral) rib parts and no sternal parts. Sternal rib parts should be absent because of the hypothesized caudal shift of the abaxial domain, which provides necessary and permissive signalling for the sternal rib parts (Figure 2). For *Choloepus*, the prediction is that the first seven vertebrae will not have ribs, like almost all other mammals. The hypothesized forward shift of the abaxial domain cannot lead to the induction of ribs or parts of ribs in cervical vertebrae, as abaxial signalling for sternal rib parts is only permissive and not instructive (as discussed above). Hence, the prediction for *Choloepus *is of a normal mammalian pattern of seven cervical vertebrae.

#### Transitional vertebrae and rudimentary ribs

**Figure 4:**
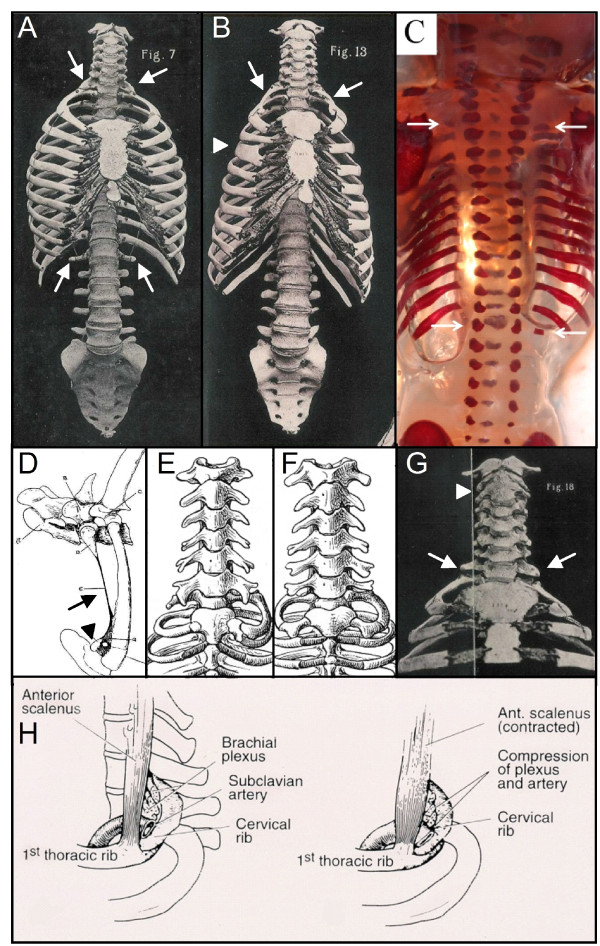
**Homeotic transformations in humans and other mammals**. A) Human skeleton with rudimentary ribs on the 7th and 19^th ^vertebrae, indicating incomplete homeotic transformations at the cervico-thoracic and thoraco-lumbar boundary (white arrows). Note the change from 24 to 23 presacral vertebrae. From [81]. B) Human skeleton with rudimentary ribs on the first thoracic vertebra (white arrows). Note the change from 24 to 25 presacral vertebrae, the abnormal shape of the fourth rib on the right (white arrowhead) and the asymmetric sternum (asymmetric transition of the manubrium to the corpus sterni). From [81]. C) Human fetal skeleton with rudimentary ribs on the 7th and unilaterally on the 19^th ^vertebrae (white arrows). From [85]. D) Rudimentary first rib with long fibrous band (arrow) connecting to the first rib and sternum in horse (cf. *Trichechus manatus *with rudimentary rib and fibrous band in Figure 7F. Note that the sternal part is present, attached to the sternum (arrowhead). From [78]. E) and F), unilateral and bilateral complete rudimentary ribs in the slow lori (*Nycticebus sp*.). From [5]. G) Human skeleton showing rudimentary ribs on the eighth vertebra (white arrows) and a fusion of the second and third vertebra (white arrowhead). From [81]. H) The presence of a cervical rib leads to pressure on the nerves and arteries that go into the arm, especially when the anterior scalenus muscle is contracted. This may lead to Thoracic outlet syndrome. From [139].

The homeosis hypothesis predicts that vertebrae at boundaries may have a transitional identity regarding shape and the presence of ribs as a result of incomplete homeotic transformations. This follows from results on homeotic mice mutants and is supported by skeletal patterns in other mammals with homeotic transformations of vertebrae, including humans. Homeotic transformations induced by mutations of *Hox *genes, or genes upstream of *Hox *usually appear to be incomplete, resulting in transitional vertebral identities (Figure 3) [39,66-76]). For instance, a transitional cervico-thoracic vertebra is characterised by an intermediate cervico-thoracic shape and the possession of rudimentary ribs. Rudimentary ribs can consist of only proximal parts, or proximal and medial parts or even of only proximal and sternal parts, possibly with the medial parts as fibrous bands (Figure 3A,C,D,G and 4D) [4,77-80]. It is important to note that fibrous bands cannot be seen on radiograph, nor in cleared and stained specimens and are also rarely preserved on museum skeletons. Rudimentary ribs are often fused to the adjacent rib (Figure 3D and 4F,H) or, when very short to the transverse process, leading to an enlarged transverse processes (Figure 4A and 4D) [43,81]. It is thought that incomplete homeotic transformation of vertebrae and ribs are common, because the identities of vertebrae are influenced by a set of partially redundant *Hox *genes [e.g. [13,14,16,18,82]]. Hence, a mutation in only one gene may not lead to a complete homeotic transformation, and generally further mutations are expected to be necessary for complete transformations.

The primaxial/abaxial hypothesis does not predict intermediate identities of vertebrae, other than the presence of proximal and medial rib parts without sternal rib parts on the 8^th ^and 9^th ^vertebrae in *Bradypus*, due to a caudal shift of the abaxial domain. No rudimentary ribs are predicted for *Choloepus*. No intermediate shapes of vertebrae are expected, because the mechanism is supposed not to be centered on the vertebrae, but to impact rib formation from a distance and, hence a more gradational mechanism is expected to have but little influence at the vertebral shape, which is supposedly determined by a different mechanism.

#### Homeotic transformations at other boundaries

The homeosis hypothesis predicts that homeotic changes at the cervico-thoracic boundary may be accompanied by homeotic changes at other boundaries. This follows from results on homeotic mice mutants, in particular mutations in genes upstream of *Hox *and compound *Hox *mutations (Figure 3F) [14,18,39,69,73,74,76,83]. A shift at several boundaries is also seen in skeletal patterns in other mammals with homeotic transformations of vertebrae, including humans (Figure 4A,C) [e.g. [10,81,84,85]]. Hence, the homeosis hypothesis suggests the possibility of transitional and asymmetric vertebrae at other vertebral boundaries, e.g. lumbar vertebrae with rudimentary ribs. A homeotic shift that includes the lumbo-sacral boundary implies a shift of the sacrum, a change of the number of presacral vertebrae and the possibility of sacral vertebrae that are incompletely fused to the sacrum (Figure 3F).

The primaxial/abaxial hypothesis predicts no homeotic transformations of vertebrae at other vertebral boundaries. It also does not predict a shift in the presence of ribs at the thoraco-lumbar boundary. The primaxial/abaxial hypothesis predicts a shift of the sacrum and sites of sacral fusion along the vertebral column, leading to a change in the presacral number of vertebrae, just as the homeosis hypothesis.

#### Left-right asymmetry of vertebrae and ribs

**Figure 5:**
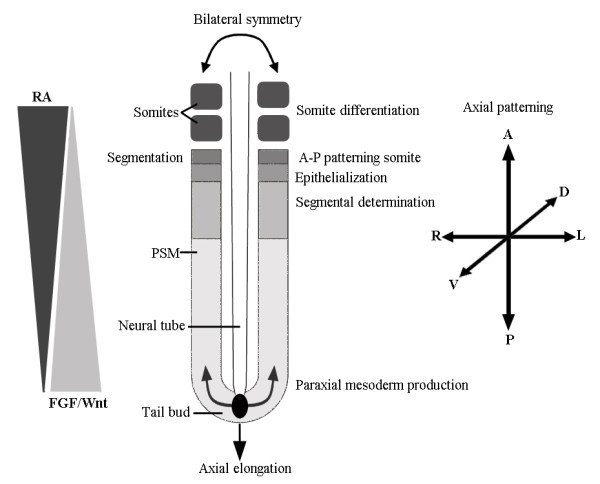
**Opposing A-P gradients of Retinoic acid (RA), Fgfs and Wnts during early organogenesis influence most processes that take place during this stage, including A-P, Medio-Lateral and Dorso-Ventral patterning of the three germ-layers of the embryo, axial lengthening, cell migration, somitogenesis and the active maintenance of bilateral symmetry of the left and right somites**. Modified with permission from [139]

The experiments with mutant mice indicate that vertebrae with a transitional identity are usually asymmetric, e.g. with asymmetric shape, with one rudimentary rib larger than the other, or with a unilateral rib (Figure 3A,G). This squares with the asymmetry in transitional vertebrae in other mammals, including humans (Figure 4C). The asymmetry in vertebrae with a transitional identity is presumably due to a coupling between A-P patterning of presomitic mesoderm and the preservation of left-right symmetry of somite formation. Both processes are to an important extent determined by the same A-P gradients of Retinoic acid, Fgfs and Wnts during early organogenesis (Figure 5) [[20,25,26] see also ref. [10] on unilateral cervical ribs in humans]. Hence, a modification of these gradients is expected to affect both the A-P patterning of the presomitic mesoderm and the left-right symmetry of somites. The homeosis hypothesis, thus, predicts that transitional vertebrae will often be asymmetric regarding shape and the possession and size of ribs.

The primaxial-abaxial hypothesis does not predict left-right asymmetry in the shape of the vertebrae, however if the abaxial domains are not shifted to the same extent on the left and right side, asymmetry in the possession of ribs is expected and asymmetry in the fusion of sacral vertebrae to the sacrum.

#### Pleiotropic effects

The homeosis hypothesis predicts that changes in the number of cervical vertebrae will almost always be associated with pleiotropic effects as earlier documented in humans [[10,30], see also [9,43,47,48,86-91]]. The unavoidability of pleiotropic effects is assumed to be due to the strong interactivity between the patterning of the A-P axis (vertebra identity), the dorso-ventral axis and the left-right axis during early organogenesis (Figure 5). By extension, the homeosis hypothesis predicts a wide variety of pleiotropic effects to be associated with homeotic transformations of cervical vertebrae in sloths.

Primaxial/abaxial patterning of the migrating sternal rib cells occurs after the highly interactive early organogenesis stages (in mice after E.D. 11, see [92]), when development has become more modular and compartmentalized and mutational changes with an effect on these stages are associated with fewer pleiotropic effects [27,31]. Hence, changes affecting primaxial/abaxial signaling may be expected to be primarily associated with only local pleiotropic effects in neighbouring structures. In agreement with this, Buchholtz and Stepien [56] argue that potentially deleterious pleiotropic effects, such as found in humans with cervical ribs, are not expected in sloths.

### Testing the predictions

#### Number of cervical vertebrae

Comparison of the shape of the most rostral vertebrae of wild-caught *Choloepus *and *Bradypus *specimens with those of other mammals confirms the traditional view that sloths have an abnormal number of cervical vertebrae. In the investigated *Choloepus hoffmanni *specimens, we found that the four or five most anterior vertebrae have no ribs and a cervical shape, with bilaterally a foramen transversarium, spinous processes with a dorsal and slightly caudal orientation and transverse processes with a lateral and somewhat ventro-caudal orientation (Figure 1C). The anterior tuberculi, that normally characterize the sixth vertebra in mammals were present on the fourth or fifth vertebra, indicating a homeotic transformation. The sixth vertebra had a transitional cervico-thoracic shape and rudimentary ribs. The seventh vertebra had a completely thoracic shape (Figure 1C) and full ribs that were fused to the sternum (Table 1). Thoracic shape characteristics included dorsolaterally oriented transverse processes, posteriorly oriented spinous processes and rib articulation facets. In the investigated *Choloepus didactylus *specimens, we found that the five or six most anterior vertebrae had cervical shape characteristics and no ribs (figure 1A and 1B). The seventh vertebrae always had a transient cervico-thoracic shape and rudimentary ribs (figure 1A and 1B, Table 1) and never a completely thoracic shape as in *Choloepus hoffmanni*. In most of the investigated *Bradypus *specimens, the first eight vertebrae had a fully cervical shape with bilaterally a foramen transversarium and the largest tuberculi anterior on the eighth vertebra instead of on the sixth as in other mammals (figure 1C). In two specimens, the eighth vertebra had a transitional cervico-thoracic shape (table). In all investigated specimens the ninth vertebrae had transitional cervico-thoracic shape characteristics and rudimentary ribs (Figure 1C,D, Table 1). The tenth vertebrae were in all cases the first fully thoracic vertebrae with complete ribs that were fused to the sternum, comparable to the eighth vertebrae in other mammals.

Hence, the shapes of the vertebrae and the presence or absence of ribs support that homeotic transformations of vertebral identity have taken place in sloths, resulting in a larger number of cervical vertebrae in *Bradypus *and a smaller number of cervical vertebrae in *Choloepus *(cf. Figures 1 and 2).

Clearly, our findings do not support the primaxial/abaxial hypothesis. This hypothesis can only explain the absence of sternal rib parts on the 8^th ^and 9^th ^vertebrae in *Bradypus*, i.e. rudimentary ribs that consist of the proximal and medial rib parts. However, the completely missing ribs on the eighth cervical vertebrae in *Bradypus *cannot be explained, nor the rudimentary ribs on the ninth that only consist of the proximal part of the rib (Figure 1C,D cf Figure 2). Similarly, the presence of rudimentary ribs on the sixth and full or rudimentary ribs on the seventh cervical vertebrae in *Choloepus *are not predicted by the primaxial/abaxial hypothesis (Figure 1A,B cf Figure 2). Buchholtz and Stepien [56] assume that such rudimentary ribs can be explained by the primaxial/abaxial hypothesis, but this would not only require instructive abaxial patterning of these rib parts and the development of isolated sternal rib parts, but also the unlikely articulation or fusion of sternal rib parts to the vertebrae, far away from the sternum. The latter would require the migration of future sternal rib cells away from the vertebrae towards the developing sternum to receive abaxial signalling, followed by the migration away from the sternum back towards the vertebrae. Finally, none of the shape changes of cervical and thoracic vertebrae in *Bradypus *and *Choloepus *was in agreement with the hypothesis.

#### Transitional vertebrae at other vertebral boundaries

**Figure 6:**
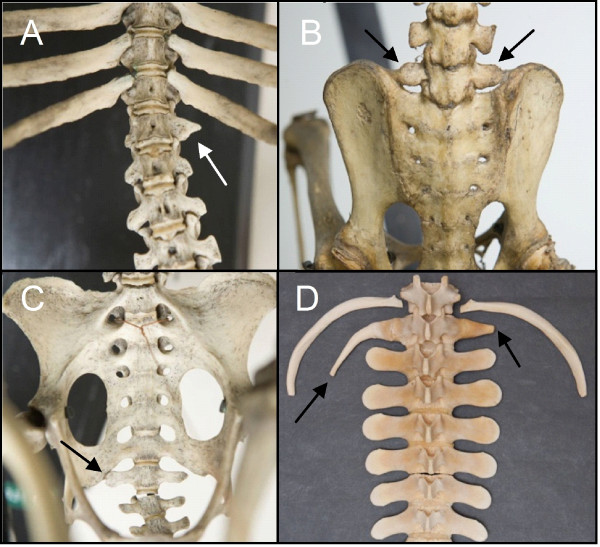
**Transitional vertebrae in sloths and manatees showing incomplete and asymmetric transformations**. A) Ventral view of a transitional thoraco-lumbar vertebra of a *Bradypus tridactylus *(ZMA.331) with a short rudimentary rib on the left (white arrow). B)Dorsal view of a transitional lumbo-sacral vertebra of a *Choloepus didactylus *(ZMA.334) with bilaterally incomplete fusion with the sacrum (arrows). C) Ventral view of a transitional sacro-coccygeal vertebra in a *Bradypus tridactylus *(ZMA.331) with on the right incomplete fusion with the sacrum (arrow). D) ventral view of a transitional thoraco-caudal vertebra in a *Trichechus manatus *(RMNH.MAM.22392),with a rudimentary rib on the right side and a transitional transverse process without rib on the left side (arrows).

As already mentioned above, the vertebrae at the cervico-thoracic boundary often had a transitional identity in *Bradypus *and *Choloepus*. The vertebrae at other vertebral boundaries also often had a transitional identity. At the thoraco-lumbar boundary transitional vertebrae had a transitional shape and rudimentary ribs (Figure 6A). At the lumbo-sacral and sacro-coccygeal boundaries, incomplete sacral fusions indicate transitional vertebral identities (Figure 6B,C). These transitional identities are in agreement with the predictions of the homeosis hypothesis.

The primaxial/abaxial hypothesis does not predict the observed rudimentary lumbar ribs in *Bradypus*. The rudimentary lumbar ribs in *Choloepus *are also not in agreement with this hypothesis, because they are not only missing the sternal parts, but also the medial parts of the ribs. The primaxial/abaxial hypothesis cannot explain the transitional shape characteristics of vertebrae at the lumbo-sacral and sacro-coccygeal boundaries which rather point to incomplete homeotic transformations. Incomplete and asymmetrical fusions at these boundaries are not in agreement with this hypothesis, because they were associated with transitional shape characteristics of the involved vertebrae.

#### Left-right asymmetry

Vertebrae with a transitional identity usually had a strong left-right asymmetry in *Bradypus *and *Choloepus *(figures 1C, 6A,C). This was true for vertebrae at all vertebral boundaries and could be apparent both in the shape of the vertebrae, the size and shape of the ribs and the extent of the fusion with the sacrum. The asymmetry is predicted by the homeosis hypothesis and only for the presence of ribs for the primaxial/abaxial hypothesis.

#### Pleiotropic effects

**Figure 7:**
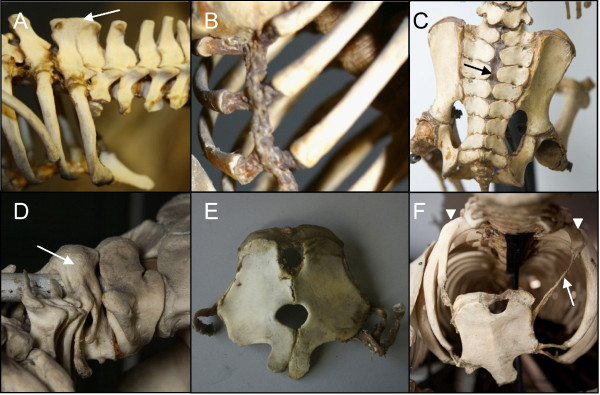
**Skeletal and fibrous abnormalities in sloths and manatees**. A) Fusion of the dorsal spinous processes of the seventh and eighth vertebra in a *Choloepus didactylus *(RMNH.MAM.7203) (arrow). B) Absence of ossification in the sternum of a *Choloepus didactylus *(ZMA.335). C) Defective ossification of the sacrum in a *Choloepus didactylus *(ZMA.335) (arrow). D) Fusion of the second and third vertebra in a *Trichechus senegalensis *( ZMA14042) (white arrow). E) large sternal foramen, a midline fusion defect, in a *Trichechus manatus *(RBINS 1.181). F) Abnormal fibrous band in a *Trichechus manatus *(U. Nat coll) (white arrow) that connects the rudimentary rib on the left with the sternum. Note the asymmetry of left and right ribs (white arrowheads). The position of the fibrous band suggests that the mesenchymal anlage of the rib was formed, but that there was a cell fate change from chondrification (and later ossification) to the formation of a fibrous band, see text and cf horse with rudimentary first ribs and fibrous band in Figure 4D.

We found many abnormalities in the skeletons of *Choloepus *and *Bradypus *specimens, affecting vertebrae, ribs, sternum, cranium, pelvic girdle and limb bones (Figure 7, Table 1). Fusions of cervical vertebrae were found to be quite common, in particular of the second and third vertebrae, but also of other cervical vertebrae (Figure 7A). Fusions of vertebrae can be caused by segmentation defects during early development due to the link between segmentation and A-P patterning (Figure 5) or by abnormal fusion, which can be due to increased ossification at a later stage. Furthermore, we found defective chondrification and ossification of the sternum and pelvic girdle in three *Choloepus *specimens (Figures 7B,C, Table 1). Abnormal fibrous bands were found in two *Choloepus *specimens that connect rudimentary ribs with the sternum and apparently had developed from the anlagen of medial and distal rib parts instead of the normal bony parts [see [93]]. Other abnormalities were asymmetric vertebrae and ribs, presumably caused by a disturbance of the preservation of left-right symmetry of developing somites [25,26]. These abnormalities in *Choloepus *and *Bradypus *specimens are in agreement with the predictions of the homeosis hypothesis In contrast, the primaxial/abaxial hypothesis does not predict this wide- array of skeletal abnormalities in different parts of the body, especially not the observed disturbances in chondrification and ossification and possibly segmentation.

## Skeletal patterns in sloths, manatees and their sisterspecies

### Similarities beween sloths and manatees

#### Vertebral homeotic transformations

Manatees, like Choloepus, have a reduced number of cervical vertebrae, based on shape characteristics and the number of anterior vertebrae without ribs (Figure 1E,F). Comparison of skeletons of wild-caught manatees (*Trichechus manatus *and *T. senegalensis*) with those of sloths showed a surprisingly high similarity. Manatees also typically displayed incomplete homeotic transformations at the cervico-thoracic and other vertebral boundaries: they usually had rudimentary ribs anterior to the first full ribs and the shape of vertebrae at the cervico-thoracic boundaries was often transitional (Figure 1E, Table 2). At the thoraco/caudal boundary vertebrae also often displayed a transitional character in shape and in presence of ribs (Figure 6D). More caudally it was difficult to distinguish clear vertebral boundaries, due to the reduction of the pelvic girdle and the absence of sacral fusions. Another similarity with sloths was the strong left-right asymmetry of transitional vertebrae, e.g. regarding size and presence of foramina transversaria (cervico-thoracic boundary), size of transverse processes (thoraco-caudal boundary) and size of rudimentary ribs (cervico-thoracic and thoraco-caudal boundary). The seventh vertebra of the investigated manatee specimens always had a full thoracic identity (Figure 1E,F, Table 2). The sixth cervical vertebrae were transitional with rudimentary ribs and often missed foramina transversaria (1E). Sometimes the fifth cervical vertebra also had rudimentary ribs (always smaller than those on the sixth).

#### Pleiotropic effects

We found many abnormalities in the skeletons of manatees (Table 2, Figure 7). Particularly frequent, like in *Choloepus *were fusions of the second and third cervical vertebrae (Figure 7D, Table 1). In *Choloepus *and *Bradypus *we found abnormally shaped and asymmetric vertebrae, ribs and sterna (Figure 7D). Midline fusion defects of the sternum were particularly common (Figure 7E), as well as reduced and unfused spinous processes of cervical vertebrae (Figure 7D). Finally, we also found large abnormal fibrous bands, including fibrous bands that connected rudimentary ribs to the sternum (Figure 7F). Hence, several skeletal abnormalities were remarkably similar to those found in sloths.

### Skeletal patterns of related taxa

#### Armadillos and anteaters

We investigated the vertebral pattern in 27 wild-caught armadillos (mainly *Euphractus sexcinctus *and *Dasypus novemcinctus*, see Table 4) and 8 wild-caught collared anteaters (*Tamandua tetradactyla*), sister taxa of the sloths within the Xenarthra. In armadillos and all but one collared anteater, the number of cervical vertebrae was found to be seven, as is typical for mammals (Table 3 and 4). We did find several transitional vertebrae at the thoraco-lumbar boundary with rudimentary ribs and at the lumbo-sacral boundary with incomplete sacral fusion. Transitional sacro-coccygeal vertebrae were quite common.

We did not find skeletal abnormalities in these specimens, other than the above-mentioned transitional vertebrae and irregulaties in tail vertebrae in some collared anteaters (Table 3). The transitional vertebrae commonly displayed left-right asymmetry, as in sloths and other mammals. Postnatal fusions of cervical vertebrae are typical for armadillos and supposedly adaptations to their digging habits [94,95]

One anteater specimen was found to have rudimentary ribs on the seventh cervical vertebra and it had unilaterally one tuberculum anterior on the fifth cervical vertebra and one on the sixth vertebra, indicating incomplete homeotic transformations of C5, C6 and C7 (Table 3). This specimen was found to have incomplete ossification of the sternum and a phalanx missing in digits 1 and 2 of the left hindlimb (oligodactyly).

#### Dugons and hyraxes

**Figure 8:**
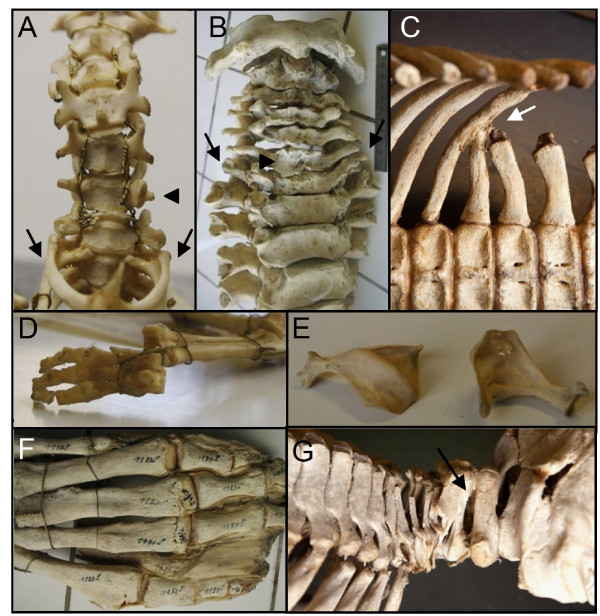
**Skeletal and fibrous abnormalities in individuals of Dugongs and hyracoids with an aberrant number of cervical vertebrae**. A) *Dendrohyrax arboreus *(RMCA 22057) skeleton with full ribs on the seventh vertebra (arrows) and a sixth cervical vertebra with on the right the identity of a normal seventh vertebra and on the left of a normal sixth vertebra (with anterior tuberculum, arrowhead). B) *Dugong dugon *(RBINC 1.183d) with articulation facets for rudimentary ribs on the seventh cervical vertebra (arrows). Note the irregular shape of the cervical vertebral bodies, in particular of the sixth one (arrowhead). C) *Dugong dugon *(RMNH.MAM.27523) with a small rudimentary rib on the first caudal vertebra that is fused to the last thoracic rib (arrow). Note that the transverse process of the second caudal vertebra is fused to the rudimentary rib. D) Forefoot of a *Dendrohyrax arboreus *(RMCA 22057) showing four instead of five digits. Same specimen as in A) with full cervical ribs. E) Abnormally shaped scapulae of a *Procavia capensis *(RMCA 20098) with rudimentary cervical ribs. F) Abnormal ossification of tendons in the limb of a *Dugong dugon *(RBINC 1.183d) with rudimentary cervical ribs (same specimen as B). G) *Dugong dugon *(RMNH.MAM.27523)with fused second and third vertebrae (arrow) with a unilateral rudimentary rib (not shown).

We investigated 11 wild-caught specimens of dugongs (*Dugong dugon*), a sister taxon of manatees within the order Sirenia. Unexpectedly, we found in approximately half of the specimens a reduced number of cervical vertebrae (6 of 11, Table 5). These speciments all had 6 cervical vertebrae and rudimentary ribs on the seventh vertebra, signifying a transitional cervico-thoracic identity, Figure 8B). All of these specimens had skeletal abnormalities, including irregularly shaped and asymmetric vertebrae, ribs and sternums, fusions of cervical vertebrae and ribs, abnormalities in limbs and scapula, and midline fusion defects in the skull (Figure 8C,F,G). We did not find skeletal abnormalities in those specimens that had the normal number of seven cervical vertebrae.

Furthermore, we investigated 16 wild-caught specimens of hyraxes (Procaviidae), a sister taxon of the Sirenia within the Paenungulata (*Dendrohyrax arboreus, D. dorsalis *and *Procavia capensis*). We found one *D. arboreus *and one *P. capensis *with 6 cervical vertebrae and rudimentary or complete ribs on the seventh cervical vertebrae (Figure 8A, Table 6). Both specimens had transitional thoraco-lumbar vertebrae and the *D. dorsalis *specimen also had an asymmetric sixth cervical vertebra with on the right a C6 identity and on the left a C7 identity (Figure 8A). Furthermore, both specimens had skeletal abnormalities, including irregularly shaped vertebrae and ribs, malformed scapulae and oligodactyly, Figure 8D,E, Table 6). The remaining 14 specimens had the normal number of seven cervical vertebrae with sometimes transitional vertebrae at more caudal boundaries, Table 6). We did not find any further skeletal anomalies in these specimens.

## Discussion

### Homeotic transformations of vertebrae in sloths, manatees and related taxa

#### Support for homeotic transformations in sloths

We found that the number of cervical vertebrae in *Choloepus *and *Bradypus *has changed from the standard seven of most mammals due to homeotic transformations of vertebrae, as first suggested by Bateson [34]. We conclude this based both on the shapes of the vertebrae and the absence or presence of ribs (cf. Figure 1 and 2). There was generally good agreement between the degree of transformation of vertebrae and the size of the ribs, e.g. vertebrae with full ribs displayed fully thoracic shape characteristics, whereas vertebrae with one or more rudimentary ribs displayed a transitional cervico-thoracic shape. Homeotic transformations also occurred at more caudal boundaries, as demonstrated by the presence of vertebrae with a transitional identity at these boundaries (Figure 6). The vertebral pattern with homeotic shifts along a large part of the vertebral column mostly resemble the vertebral patterns of mice with mutations in genes upstream of *Hox*, which affect multiple *Hox *genes [e.g. [69,74,76,83,96,97], see also [23]]. This is in agreement with preliminary findings of us that mutations in genes upstream of *Hox *are involved in the development of abnormal number of cervical vertebrae in humans (Bakker et al., unpublished data).

All other predictions of the homeosis hypothesis are also supported by our findings on the derived skeletal patterns of sloths. Incomplete homeotic transformations of vertebrae were common at all vertebral boundaries and transitional vertebrae often displayed strong left-right asymmetry. Furthermore, we found many skeletal and fibrous band abnormalities in *Choloepus *and *Bradypus*, similar to those found in humans and mice with a changed number of cervical vertebrae (cf. Figures 4D and 7F; [10,43,81,86-88,98] for humans, [19,66,67,72,75,76,99] for transgenic mice). This supports the hypothesis that homeotic changes of the number of cervical vertebrae in mammals are unavoidably associated with pleiotropic effects [10].

Buchholtz and Stepien [56] argue instead that homeotic transformations of cervical and thoracic vertebrae have not taken place in sloths. However, their illustrations of vertebrae of *Bradypus *and *Choloepus *clearly show similar signatures of a homeotic transformation as did our specimens, i.e. a cervical shape of the eighth vertebra of *Bradypus *with bilateral foramina transversaria, no articulation facets and a cervical orientation of the processes (their Figure 1D) and a thoracic shape of the sixth and seventh vertebrae of *Choloepus *with no foramina transversaria, the presence of articulations facets and a thoracic orientation of processes (their Figures 2C and 2F). In addition, in their Figures 2C and F the shapes of the sixth and seventh vertebrae in *Choloepus *indicate respectively a transitional cervico-thoracic identity and a completely thoracic identity. Hence, their figures strongly support homeotic transformations of the vertebrae.

##### Rudimentary ribs as homeotic transformations

Buchholtz and Stepien [56] argue that incomplete homeotic changes should lead to "anterioposterior discordances" and not to the truncated ribs that are common in sloths and that they describe as 'mediolateral discordances' (page 74 in [56]). In contrast to their claim, rudimentary (truncated) ribs are a hallmark of incomplete homeotic transformations and are therefore, best interpreted as transitionally sized [60-66,68,69,74] (see also the sub-section **Transitional vertebrae and rudimentary ribs**). In sloths and in other mammals with homeotic transformations, both short and long rudimentary ribs occur. Short rudimentary ribs in general are fused with the transverse processes of the vertebra, as in other mammals, so that it looks like an enlarged transverse process (Figure 1B and 1C, Figure 4A) [43] while larger rudimentary ribs often fuse with the adjacent rib, which is probably the cause that the first ribs in both manatees and sloths are often found to have an irregular shape [56,81].

##### Co-ordinated shift of limb plexuses

Buchholtz and Stepien (page 75/76 in [56]) furthermore argue that the rostral shift of the brachial nerve plexus in *Choloepus *and the caudal shift in *Bradypus *[100] is inconsistent with a homeotic shift of the cervico-thoracic vertebral boundary. In contrast, homeotic shifts of the cervico-thoracic and lumbo-sacral boundaries of one or more vertebrae generally co-occur with a homeotic shift of the brachial and lumbo-sacral plexuses in homeotic mice mutants [101-109]. The coordination of the homeotic shifts is thought to be due to the simultaneous and co-ordinated anterior-posterior patterning of the adjacent paraxial mesoderm and neural tissues, mediated by *Hox *genes in each tissue layer, in response to the same graded patterning signals [22,36,74,110-112]. As part of this co-ordinated process, instructive signaling from the paraxial mesoderm to the neural tissues is involved in the specification of the A-P identity of spinal motor neurons, including the specification of the lateral motor columns that innervate the limbs [[113,114], see also [115]]. Signaling from the limb mesoderm to the neural tissues is critical for later aspects of motor pool differentiation such as muscle-specific patterns of axonal innervation, but this appears to be permissive rather than instructive signalling [reviewed in [111]]. Further support for the co-occurrrence of homeotic vertebral transformations and shifts of limb plexuses comes from humans in which complete, rather than incomplete homeotic transformations of vertebrae have occurred at the cervico-thoracic and/or lumbo-sacral boundaries [e.g. [53,89,116-119]]. Incomplete transformations often do not co-occur with a simultaneous shift of the limb plexus and this may lead to pressure on the nerves (thoracic outlet syndrome, see Introduction).

##### Ossification patterns and the cervico-thoracic boundary

Hautier et al. [120] claim that the timing of ossification of the 8^th ^and 9^th ^vertebrae in *Bradypus *species, indicates a thoracic, rather than a cervical identity. However, in general in mammals, the timing of ossification of vertebral bodies and spinous processes just follows the order of the vertebrae along the anterior-posterior axis in both anterior and posterior directions (usually starts somewhere in the thoracic region and proceeds both anteriorly and posteriorly for the vertebral bodies and starts anteriorly and proceeds posteriorly for the spinous processes) and does not identify a specific vertebral region, nor the position of a specific vertebral boundary [e.g. [121-126]]. Hautier et al. [120] claim that the 8^th ^and 9^th ^vertebra have a thoracic ossification pattern, because of the supposed simultaneous ossification of the vertebral bodies of these vertebrae together with those of the other thoracic vertebrae. However, the sparse data on the relative timing of the ossification of vertebral bodies around the cervico-thoracic boundary in *Bradypus variegates *and *B. tridactylus *([120], suppl. data) suggests that ossification of this region occurs in a roughly sequential posterior-anterior order, as in other mammals: the 12^th ^vertebral body before the 11^th^, the 10^th ^before the 9^th^, the 9^th ^vertebra before the 8^th^, the 8^th ^before the 7^th ^and the 7^th ^before the 6^th ^(cf. ZMB/33812, ZMB/41122, MNHN/1881-111 and MNHN/1902-32b). Hence, like in other mammals, the ossification patterns do not indicate a cervico-thoracic boundary in *Bradypus*. The relative timing of ossification of the spinous processes of the 8^th ^and 9^th ^vertebrae in *Bradypus *cannot be determined from the data of Hautier et al. [120]. Hence, we conclude that the claim that the 8^th ^and 9^th ^vertebrae in *Bradypus *have a thoracic identity based on vertebral ossification patterns, is insufficiently supported.

Furthermore, although the timing of ossification can provide information about homologies [127], changes in the timing of ossification patterns do not necessarily reflect changes in the early specification of mesenchymal anlagen. For instance, in species in which specific bony parts are used prematurely, or differently, ossification of these parts may occur prematurely, leading to derived ossification patterns, as in moles and marsupials [127-129]. It is possible that the early ossification of anterior cervical vertebral bodies in armadillos [120], may be related to the increased ossification and fusion of cervical vertebrae in this species. Interestingly, one *Bradypus tridactylus *specimen also shows early ossification of an anterior cervical vertebra (C2, [120] suppl. data), further investigation is necessary to find out whether this is typical for the species or due to abnormal ossification of anterior cervical vertebrae in this specimen.

Finally, intraspecific variation in ossification patterns is common and even minor disturbances of development can lead to a conspicuous delay of ossification of specific skeletal elements, not necessarily leading to post-natal phenotypic changes [e.g. [126,130,131]]. In conclusion, we do not think that the data on ossification sequences in sloths can be used for evaluating the validity of the primaxial-abaxial and homeosis hypothesis in sloths.

#### No support for primaxial/abaxial repatterning in sloths

Most predictions of the primaxial/abasxial hypothesis are not supported by the vertebral anatomy of sloths. Only the absence of distal parts of ribs on the eighth and ninth vertebra in *Bradypus *can be explained by the primaxial/abaxial hypothesis, but not the absence of the medial rib parts or the complete absence of ribs (cf. Figure 1C,D with Figure 2). In addition, no other rudimentary ribs in *Choloepus *and *Bradypus *can be explained by this hypothesis, i.e. rudimentary ribs on cervical vertebrae in *Choloepus *and on lumbar vertebrae in *Bradypus*. Not only that, the cervical shape characteristics of the eighth vertebra and the cervical or cervico-thoracic characteristics of the ninth vertebrae in *Bradypus*, are in contradiction with this hypothesis and clearly demonstrate a homeotic transformation. Similarly, the cervico-thoracic or completely thoracic shape characteristics of the sixth and seventh vertebrae in *Choloepus *cannot be explained by this hypothesis nor other homeotic transformations of shapes, such as the shifted position of the largest anterior tuberculi. Finally, the primaxial/abaxial hypothesis does not predict the common left-right asymmetry of vertebrae and sternum, nor the associated skeletal abnormalities (Figures 6 and 7).

#### Homeotic transformations in manatees

Manatees also have an exceptional number of cervical vertebrae and like in sloths this appears to be due to homeotic transformations. As in sloths, we find incomplete and complete homeotic transformations of vertebrae, as apparent from the shape of vertebrae and the absence or presence of ribs. Furthermore, we find that transitional vertebrae are usually asymmetric and that homeotic transformations occur at different vertebral boundaries. Hence, we find striking similarities between the vertebral patterns in sloths and manatees. In addition, we also find that the changes of the number of cervical vertebrae in manatees co-occur with skeletal and fibrous band abnormalities.

Our results, thus, strongly support the traditional view that the number of cervical vertebrae in manatees is changed due to homeotic transformations as in sloths [6,8,41]. The results also confirm that homeotic changes of cervical vertebrae generally co-occur with pleiotropic effects in other parts of the body.

#### Comparisons with sister taxa of manatees and sloths

Most investigated specimens of armadillos, anteaters and hyraxes, have the normal number of seven cervical vertebrae. Transitional vertebrae at more caudal boundaries were found to be common, as is more generally the case in mammals (Figure 3F,G,I and Figure 4A,C) [see further [74]]. The number of vertebrae in caudal regions shows more interspecific variation in mammals [6] and, hence, caudal homeotic transformations appear to be less constrained, in agreement with an observed weaker selection against such changes in humans [10]. The transitional vertebrae were usually asymmetric, which appears to be a more general phenomenon, as asymmetric transitional vertebrae also appear to be common in *Anolis *lizards (at the thoraco-lumbar and more caudal boundaries, Andre Pires da Silva, pers. comm.). Unexpectedly, we found that 6 of 11 investigated specimens of Dugongs had a changed number of cervical vertebrae and rudimentary ribs on the seventh vertebrae (Figure 7B, Table 5). Thus, although this species is thought to have the normal number of vertebrae for mammals, it seems that the constraint on changes of the number of cervical vertebrae has also been broken in this sisterspecies of manatees, albeit to a lesser extent. In addition, we found that two out of 17 hyracoid specimens and one out of 8 anteater specimens had an abnormal number of cervical vertebrae, with rudimentary or full ribs on the seventh vertebra (Figure 8A, Table 4 and 6, Lin and Asher also found cervical ribs in some *P. capensis *specimens, pers. comm.). Hence, it is possible that the evolutionary constraint on changes of the cervical vertebral number is also weaker in these sister taxa. It will be interesting to investigate the strength of the evolutionary constraint in xenarthrans and paenungulates and possibly other afrotherians further. A first datum already is that the constraint on the number of presacral vertebrae also appears to be relaxed in afrotherians and xenarthrans [6,132]. One reason may be that biomechanical constraints on both transitional cervico-thoracic and lumbo-sacral vertebrae are weak due to relatively low activity rates of members of these taxa.

Importantly, none of the anteater, armadillo, dugong and hyrax specimens with a normal number of seven cervical vertebrae had conspicuous skeletal abnormalities, other than transitional vertebrae at caudal boundaries and minor anomalies such as irregular vertebrae in the long tail of anteaters. In contrast, all Dugong, anteater and hyracoid specimens with 6 cervical vertebrae had conspicuous skeletal abnormalities (Figure 8B to 8G), as we also find in manatees and sloths.

### Homeotic changes and fitness consequences

#### Pleiotropic effects and fitness

The skeletons of sloths and manatees that we investigated had a surprisingly high number of abnormalities, similar to those found in mice and humans with an abnormal number of cervical vertebrae. In addition, the dugong, anteater and hyrax specimens with a changed number of cervical vertebrae, also had similar skeletal abnormalities. This strongly supports the hypothesis that changes in the number of cervical vertebrae in mammals are unavoidably accompanied by pleiotropic effects [8-10]. These abnormalities were shown to lead to strong, indirect, selection against these changes in humans and we expect similarly strong selection against such abnormalities in most other mammalian species, especially in highly active mammals. However, it is plausible that skeletal abnormalities are less harmful in sloths, manatees and dugongs, because of their low activity and low metabolic rates. We cannot exclude that some of the abnormalities are also harmful for sloths, manatees and dugongs, for instance the virtually incomplete ossification of the pelvic girdle and sternum in two *Choloepus *specimens. Nevertheless, the high frequency of the abnormalities in wild-caught specimens supports the notion that fitness effects of the abnormalities appear to be limited.

#### Cancer and metabolic rate

We only investigated skeletons, hence we have no information on potential abnormalities in other tissues. Data on humans with a changed number of cervical vertebrae show that many other deleterious pleiotropic effects contribute to a lower fitness of these individuals and lead to strong prenatal and neonatal selection. In particular, in those individuals with a changed number of cervical vertebrae that survive the prenatal and neonatal period, there is a strongly increased chance for childhood cancers (120-fold, [8]). The extremely low metabolic rates of manatees and sloths are expected to lower cancer rates [9]. A similar effect would apply to dugongs, anteaters and hyraxes as these also have low metabolic rates, albeit not as extreme as in sloths and manatees [133-136] and, hence, they may also have reduced cancer rates.

## Relaxation of stabilizing selection allows breaking of constraint in sloths and manatees

We earlier proposed that relaxation of stabilizing selection is important for the breaking of pleiotropic constraints on body plan changes, including changes of the number of cervical vertebrae in mammals [8,9]. The common occurrence of skeletal abnormalities in the investigated sloths, manatees and dugongs strongly supports this hypothesis, because it implies that stabilizing selection against these supposedly pleiotropic effects must be weak. The weak selection is probably due to a lower number and lower harmfulness of pleiotropic effects, both related to the extremely low activity and metabolic rates. Low activity is expected to minimize the harmfulness of skeletal and other anatomical abnormalities, and hence, lowers the biomechanical constraint on changes of the number of cervical vertebrae. Low metabolic rates are expected to reduce the harmfulness of pleiotropic effects, in particular by reducing the incidence and severity of cancer and other free radical associated diseases, as mentioned above [49-52].

## Constraint is weaker in reptiles and birds

The strong evolutionary conservation of the number of cervical vertebrae in mammals contrasts with the high evolutionary variability for this number in sauropsids (extant reptiles, birds and their amniote ancestors). Part of the explanation for the weaker evolutionary constraint may be related to the larger number of cervical vertebrae than mammals, with some elasmosaurid plesiosaurs having even up to 76 cervical vertebrae [137]. A larger number of cervical vertebrae implies later determination of the cervico-thoracic boundary, due to the rostro-caudad formation of the somites from which the vertebrae develop. We earlier hypothesized that a late determination of the cervico-thoracic boundary, after the most vulnerable and interactive part of early organogenesis has occurred, may be less evolutionary constrained [30]. This hypothesis explains for cervical vertebrae the rule, first proposed by Geoffroy St. Hilaire and later by Darwin, that when any part or organ is repeated many times in the same individual the number is variable. We have earlier also hypothesized that a lower cancer rate in birds and reptiles may be involved in the apparently weaker evolutionary constraints on changes of the number of cervical vertebrae in these taxa (8,9,30). Other factors are probably involved, as well, and further study of cervical vertebral numbers in sauropsids, theropsids and their tetrapod ancestors is necessary to throw more light on this puzzling difference between mammals and sauropsids.

## Conclusions

We find strong support for Bateson's hypothesis [34] that the aberrant number of cervical vertebrae in sloths and manatees is due to the homeotic transformation of cervical and thoracic vertebrae. In contrast, we find no support for the alternative primaxial/abaxial hypothesis proposed by Buchholtz and Stepien [56]. In addition, intraspecific variation in the number of cervical vertebrae in other mammalian species always appears to involve homeotic transformations, as well.

In sloths, manatees and natural mammalian mutants with an exceptional number of cervical vertebrae, homeotic changes are almost always incomplete and asymmetric. The homeotic transformations that affect the cervico-thoracic boundary are often part of a homeotic shift of a large part of the vertebral column in natural mammalian mutants. In sloths and manatees this is always the case and, as such, the vertebral changes closely resemble those found in transgenic mice with mutations in genes upstream of *Hox*. The strong association between incomplete homeotic transformation and asymmetry of vertebrae is also found at more caudal boundaries and indicates a strong interaction between the A-P patterning of the paraxial mesoderm and the maintenance of symmetry of somites.

We found a remarkably high number of skeletal and fibrous abnormalities in sloths, manatees and individuals of sistertaxa with an abnormal number of cervical vertebrae. In contrast such abnormalities were not found in individuals with a normal number of cervical vertebrae. These findings support our hypothesis that there is strong indirect selection against changes of the cervical vertebral number in mammals, due to the virtually unavoidable association with deleterious pleiotropic effects (congenital abnormalities). Additionally, we conclude that in sloths and manatees, the pleiotropic effects appear to have little or no effect on fitness, presumably due to their extremely low activity and metabolic rates. Hence, we argue that relaxed selection allows the effective breaking of the pleiotropic constraints on changes of the number of cervical vertebrae in sloths and manatees, and possibly also to a lesser extent in dugongs.

A considerable number of traits of the vertebrate body plan is probably conserved as a result of strong stabilizing selection against associated deleterious pleiotropic effects, e.g. the number of eyes, ears, nasal placodes, limbs, digits, lungs, kidneys [30]. Hence, we argue that our results on sloths and manatees and their sister taxa once again emphasize the relevance of pleiotropic constraints and stabilizing selection for the conservation of body plans. Moreover, we argue for the relevance of the relaxation of stabilizing selection for the effective breaking of pleiotropic constraints by allowing the persistence of evolutionary novelties, either temporarily till the particular associated deleterious pleiotropic effects have been removed through further evolution, or more permanently as in sloths.

## Competing interests

The authors declare that they have no competing interests.

## Authors' contributions

IVL, AJB, SJVM, JVA and FG analysed the skeletal patterns, IVL, AJB and FG analysed the data and literature and formulated the predictions, IVL, JAJM and FG wrote the manuscript and IVL, JVA and FG made the figures. All authors read and approved the final manuscript.
