# Correction to: Breaking evolutionary and pleiotropic constraints in mammals: on sloths, manatees and homeotic mutations

**DOI:** 10.1186/s13227-021-00183-0

**Published:** 2021-11-22

**Authors:** Irma Varela-Lasheras, Alexander J. Bakker, Steven D. van der Mije, Johan A. J. Metz, Joris van Alphen, Frietson Galis

**Affiliations:** 1 Naturalis Biodiversity Center, Darwinweg 2, 2333 CR, Leiden, The Netherlands; 2 IIASA, Laxenburg, Austria; 3 VU University Medical Centre, Amsterdam, The Netherlands

## Correction to: EvoDevo 2011, 2:11 https://doi.org/10.1186/2041-9139-2-11

In Table 1 of this article [1], the name of the three-toed sloth, Bradypus tridactylus, should be 5 rows higher in Table 1 and start with collection nr. RMNH.MAM.21576.

The corrected Table 1 is given in this erratum.

**Table 1:** Vertebral information and congenital abnormalities in investigated mammalian specimens: Folivora (sloths)

Collection	Species	Collection No.	Sex	Vertebral formula	Presacral No.	Rud. ribs on vertebrae	Skeletal and fibrous abnormalities
Folivora (sloths)							
RBINC	*Choloepus hoffmanni*	15260^b^	F	5C 1C/T 22T 1T/L 3L 6S 1S/Cg 5Cg	32	V6, V29	C2/C3 fusion, deformation of pelvic girdle, hole in scapula
		16349^b^)	F	5C 1C/T 21T 1T/L 3L 1L/S 5S 5Cg	31.5	V6	C1/C2 fusion, hole in larynx
		16348^b^	F	5C 1C/T 21T 4L 1L/S 7S 1SCg 4Cg	31.5	V6	C2/C3 fusion
NCBN	*Choloepus didactylus*	RMNH.MAM.322^b^	F	6C 1C/T 23T 4L 7S 1S/Cg 5Cg	34	V7	Malformed first ribs, hole in scapula
		RMNH.MAM.3961	F	6C 1C/T 23T 1T/L 2L 1L/S 7S 5Cg	33.5	V7	–
		RMNH.MAM.24470	n.a.	5C 2C/T 24T 4L 7S 1S/Cg 3 + Cg	35	V6, V7	Abnormal fibrous band attached To rudimentary rib
		RMNH.MAM.24469	n.a.	5C 2C/T 23T 4L 7S 4Cg	34	V6 V7	Incomplete ossification sternum
		RMNH.MAM.3274	n.a.	6C 1C/T 22T 1T/L 4L 7S 6Cg	34	V7	–
		RMNH.MAM.3465	n.a.	5C 2C/T 23T 1T/L 3L 7S 5Cg	34	V6 V7	C2–C3 fusion, ossification absent in sternum, fusion of ribs on V6–V7
		RMNH.MAM.1002^b^	F	5C 2C/T 23T 3L 1L/S 7S 4Cg	33.5	V6 V7	C2–C3 fusion, deformation of first ribs, asymmetric sternum, fusion of rudimentary ribs on V6–V7
		RMNH.MAM.7203^b^	F	6C 1C/T 23T 4L 7S 1S/Cg + Cg	34	V7	C2–C3 fusion, V7–V8 fusion, asymmetric vertebrae, fibrous band attached To rudimentary ribs
		RMNH.MAM.2552	F	6C 1C/T 24T 3L 7S 1S/Cg 5Cg	34	V7	Malformed ribs (3 most anterior ones)
		RMNH.MAM.1673	F	6C 1C/T 24T 3L 8S 5Cg	34	V7	C3, C4, C5 fused
		ZMA335	n.a.	6C 1C/T 24T 3L 8S 4CG	34	V7	Ossification absent in sternum and sacrum
		ZMA334	n.a.	5C 2C/T 23T 4L 1L/S 9S 3 + Cg	34.5	V6	C2–C3 fusion
		ZMA.336	n.a.	6C 1C/T 1C/T 22T 4L 8S 4Cg	34	V7 V8	Abnormal fibrous band attached to rudimentary rib, incomplete ossification
		RMNH.MAM.11417	F	–	–	–	Malformed humeri, oligodontia
		RMNH.MAM.1156	n.a.	1L 1L/S 7S 4Cg	–	–	^a^
		ZMA.9765	F	6S 1S/Cg	–	–	^a^
	*Bradypus tridactylus*	RMNH.MAM.21576	n.a.	8C 1C/T 15T 3L 1L/S 6S 9Cg	28	V9	Asymmetric cranium
		RMNH.MAM.21581	n.a.	7C 2C/T 14T 1T/L 3L 7S 8Cg	27	V8 V9 V24	–
		RMNH.MAM.24440	n.a.	8C 1C/T 16T	–	–	Asymmetric cranium
		RMNH.MAM.10460	F	8C 1C/T 15T 4L 6S 8Cg	28	V9	Irregularly shaped first rib
		RMNH.MAM.10459	F	8C 1C/T 14T 3L 1L/S 6S 6C 6 + Cg	27	V9	Metacarpal, metatarsal anomalies, many fractures, hole in cranium (unknown cause)
		RMNH.MAM.18781	F	8C 1C/T 14T 4L 6S 8Cg	27	V9	–
		RMNH.MAM.24421	n.a.	8C 1C/T 15T 4L 5S 10Cg	28	–	–
		ZMA331	n.a.	7C 2C/T 14T 1T/L 4L 6S 1S/Cg 7Cg	28	V8 V9 V24	Irregularly shaped vertebrae and first rib
		ZMA332	n.a.	8C 1C/T 15T 1T/L 3L 6S 1S/Cg 6 + Cg	28	V9 V25	–
		ZMA924^b^	n.a.	8C 1C/T 15T 3L 6S 7Cg	27	V9	Irregularly shaped vertebrae and first rib
		U. ZMA coll.^b^	n.a.	8C 1C/T 15T 4L 5S 1S/Cg 8Cg	28	V9	–

^a^Skeleton largely absent

^b^Died in captivity

*n.a.* not available, *C* cervical, *T* thoracic, *L*  lumbar, *S* sacral, *Cg* coccygeal, *Cd* caudal (post thoracic), *V* vertebra, *rud.* rudimentary, *NCBN* NCB naturalis, *RMCA* Royal Museum for Central Africa Tervuren, *RBINC* Royal Belgian Institute of Natural Sciences Brussels, *NHM* Natural History Museum London
